# Design and Evaluation of Dissolving Microneedles for Enhanced Dermal Delivery of Propranolol Hydrochloride

**DOI:** 10.3390/pharmaceutics13040579

**Published:** 2021-04-19

**Authors:** Jingjing He, Zichen Zhang, Xianzi Zheng, Lu Li, Jianping Qi, Wei Wu, Yi Lu

**Affiliations:** 1 Key Laboratory of Smart Drug Delivery of MOE, School of Pharmacy, Fudan University, Shanghai 201203, China; 18211030016@fudan.edu.cn (J.H.); 19211030017@fudan.edu.cn (Z.Z.); 19211030024@fudan.edu.cn (X.Z.); 20211030026@fudan.edu.cn (L.L.); qijianping@fudan.edu.cn (J.Q.); wuwei@shmu.edu.cn (W.W.); 2 Center for Medical Research and Innovation, Shanghai Pudong Hospital, Fudan University Pudong Medical Center, Shanghai 201399, China

**Keywords:** microneedles, propranolol hydrochloride, dermal delivery, hyaluronic acid, polyvinyl pyrrolidone, infantile hemangioma

## Abstract

Oral propranolol hydrochloride has been the first-line treatment for infantile hemangioma (IH), whereas systemic exposure to propranolol has the potential of causing serious adverse reactions. Dermal delivery of propranolol is preferable due to high local drug concentration and fewer adverse effects. However, propranolol hydrochloride (BCS class I) is highly hydrophilic and has difficulty in penetrating the stratum corneum (SC) barrier. Dissolving microneedles (MNs) are an efficient tool for overcoming the barrier of the SC and enhancing dermal drug delivery. In this study, propranolol hydrochloride-loaded dissolving MNs were fabricated by using hyaluronic acid and polyvinyl pyrrolidone as matrix materials. Controllable drug loading in needle tips was achieved by a two-step casting procedure. The needles were good in mechanical strength for penetrating the SC while presented excellent dissolving capability for releasing propranolol hydrochloride. In comparison with the solution counterpart, irrespective of being applied to intact skin or solid MNs-pretreated skin, dissolving MNs significantly increased the permeability and skin retention of propranolol. In conclusion, dissolving MNs could be a potential approach for enhancing dermal delivery of propranolol to treat IH.

## 1. Introduction

Dermal delivery is a preferable drug administration method for treating diseases localized within the skin due to its advantages of higher local drug concentration, lower systematic exposure, absence of hepatic first-pass metabolism, and better patient compliance [1]. However, the barrier function of the stratum corneum (SC) prevents the entry of the majority of therapeutics into the skin [2], particularly the hydrophilic ones [3,4]. A myriad of physical devices and approaches, such as microneedles (MNs) [5,6,7], iontophoresis [8,9,10], sonophoresis [11,12,13], magnetophoresis [14,15,16], electroporation [17,18,19], and photomechanical wave [20,21,22], have been developed to facilitate cutaneous penetration. Microneedles, an array of micron-sized needles, are the most effective because they enable breaking the barrier of the stratum corneum for delivery of therapeutics into the skin [23,24]. In addition, the needles are only 0.2–1.5 mm in length, producing minimal invasion and enabling self-administration like a patch [25,26].

So far, four types of MNs have been created, i.e., solid, hollow, coated, and dissolving MNs [27]. The first three types are made from silicon or metals. The fracture of the needles may cause severe damage to the skin [28]. Dissolving MNs consist of water-soluble matrix materials such as polyvinyl pyrrolidone (PVP), hyaluronic acid (HA), maltose, dextran, albumin, and chondroitin sulfate, while drugs are dispersed or dissolved in the needles [6,28,29]. Following insertion into the skin, the needles dissolve upon imbibing water from the interstitial fluids and release the drug payloads. Therefore, drugs are directly delivered into the skin to exert therapeutic effects [30], while no sharp needles are left behind [31,32]. Moreover, the manufacture of dissolving MNs is comparatively easier than other types of MNs. Dissolving MNs have been developed to deliver a series of drugs, including small molecules and macromolecules, such as DNA and protein [33,34,35].

Propranolol hydrochloride is a nonselective β-adrenergic receptor blocker, initially introduced to treat arrhythmias [36]. In 2008, oral delivery of propranolol hydrochloride was found to be highly effective for the treatment of infantile hemangiomas (IH), a common tumor of blood vessels in skin that usually occurs in infancy [37]; it then became the first-line medication. However, systemic exposure to propranolol hydrochloride may induce changes in sleep patterns, acrocyanosis, and gastrointestinal symptoms, and even serious adverse reactions including symptomatic hypotension, hypoglycemia, and bradycardia [38,39]. In contrast, dermal application of propranolol hydrochloride generates less untoward effects compared with the oral route for treating superficial hemangiomas, benefiting from high local drug concentration and less systemic exposure [40,41,42]. However, propranolol hydrochloride (BCS class I) has low transdermal transport efficiency due to its high hydrophilicity [43]. Many attempts have been made for developing preparations of propranolol aimed at enhancing the dermal permeation of propranolol [44,45,46]. Dissolving MNs could pierce the SC and directly deliver the drug into skin with little or no pain, which is an effective tool for enhanced dermal drug delivery [47,48]. Nevertheless, traditional dissolving MNs carry drugs in whole needles, inevitably leading to a waste of medicine due to the needles not being fully inserted into skin [49,50]. Hence, dissolving MNs were designed in this study to enhance skin delivery and retention of propranolol hydrochloride. Meanwhile, the drug was concentrated in the tip of the needles via a two-step casting procedure to avoid drug waste during preparation and application. Having the therapeutics confined in the tip of the needles can minimize drug loss, control penetration depth, and ensure drug loading [51].

In this study, a two-step casting procedure was proposed for the fabrication of dissolving microneedles for loading propranolol hydrochloride in needle tips. HA and PVP-K90 were utilized as the matrix materials of the needles. The MNs were characterized for their mechanical properties, morphology, drug release profiles, and skin penetration. The puncture performance and skin recovery after MN treatment were also assessed. IVIS was used to evaluate the retention profile of the drug in the skin. Additionally, skin permeation and retention of propranolol hydrochloride were quantified.

## 2. Materials and Methods

### 2.1. Materials

Hyaluronic acid (HA, Mw = 10 kDa) was provided by Furuida Biotechnology Co., Ltd. (Shandong, China). PVP-K90 was obtained from Shengpu New Materials Co., Ltd. (Shanghai, China). Propranolol hydrochloride was obtained from Yuancheng Technology Co., Ltd. (Hubei, China). Polydimethylsiloxane (PDMS) MNs mold was supplied by Prof. Wu, C.B. at College of Pharmacy, Jinan University. Rhodamine B and fluorescein isothiocyanate isomer I (FITC) were provided by Sigma-Aldrich Co. (Darmstadt, Germany). Sodium chloride (NaCl), ammonium metaphosphate (Na_2_HPO_4_), potassium dihydrogen phosphate (KH_2_PO_4_), lauryl sodium sulfate, and chloral hydrate were obtained from Sinopharm Chemical Reagent Co., Ltd. (Shanghai, China). Deionized water was produced by a Milli-Q purification instrument (Millipore, MA, USA). All other reagents were of analytical grade. Full thickness Bama porcine skin was purchased from Kaikai Technology Trade Co., Ltd. (Shanghai, China). Male SD rats weighing 150 ± 10 g were obtained from Shanghai SLAC Laboratory Animal Co., Ltd. (Shanghai, China). All animal experiments were implemented under the approval of Institutional Animal Care and Use Committee at School of Pharmacy, Fudan University. Rules outlined in the Declaration of Helsinki for all human and animal experimental investigations were complied with.

### 2.2. Preparation of Blank and Drug-Loaded Dissolving MNs

**Figure 1:**
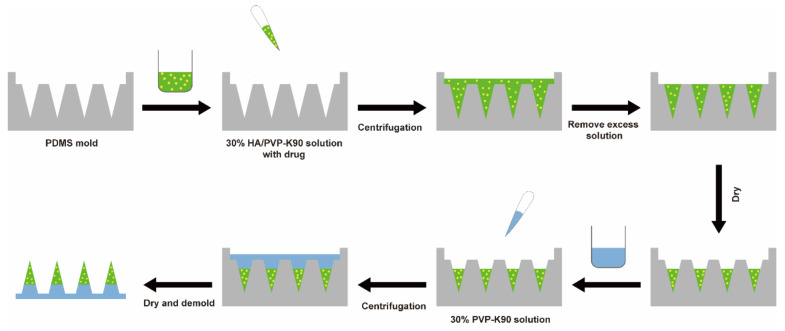
Schematics of the fabrication process of propranolol hydrochloride loaded MNs.

Micromolding is the most common strategy for the fabrication of MNs [52,53,54]. The MNs were fabricated by a micromolding method using HA and PVP-K90 via a two-step casting procedure (Figure 1) [55]. Briefly, the tip solution was prepared by dissolving HA and PVP-K90 with a weight ratio of 1:1 in distilled water to a concentration of 30% (*w/w*). The backing solution was prepared by PVP-K90 with the concentration of 30% (*w/w*). The tip solution of 200 μL was poured into the PDMS mold, which was centrifuged at 4000 rpm for 5 min to depress the solution into the PDMS mold cavities. After removing the excess solution, the PDMS mold was kept in the silica gel desiccator overnight to facilitate drying. Subsequently, 400 μL backing solution was added into the PDMS mold and then centrifuged at 4000 rpm for 10 min. The molds were put in the desiccator and dried for 24 h. Finally, the dissolving MNs were gently removed from the molds and preserved in the desiccator. In this experiment, PDMS molds could be reused in the MN production process to improve fabrication efficiency [56,57].

For the convenience of characterization and evaluation, methylene blue and FITC were used as model drugs to add in the tip matrix solution when needed and were then cast into the mold to fabricated MNs as described above.

To load propranolol hydrochloride in MNs, propranolol hydrochloride was added to the tip solution, and the final concentration of propranolol hydrochloride in the tip solution was controlled at 40, 50, 60, 70, and 80 mg/mL, then cast into the mold as described above.

### 2.3. Morphology of MNs

The morphology of MNs was observed by field emission scanning electron microscope (JEOL Ltd., Tokyo, Japan), while the drug distribution of the MNs was observed by inverted fluorescence microscope (OLYMPUS Ltd., Tokyo, Japan) with the aid of methylene blue and FITC. The height, needles pitch, and width of MNs base were evaluated.

### 2.4. Mechanical Property of MNs

The mechanical property of the MNs was measured by a TA-XT2i Texture Analyzer (Stable Micro Systems, Haslemere, UK) in compression mode as in a previously reported method [47]. The test distance was set at 900 μm with the trigger type at auto force. The pre- and post-test speed was 1 mm/s, while the test speed was 0.1 mm/s. The trigger force was set at 0.02 N. The tips of the MNs were kept upward, which were pressed by an axial compression force via the texture analyzer. The force was then analyzed to obtain a force-displacement curve, and the failure force of MNs was recorded as the needles began to break. Finally, the morphology of the compressed MNs was observed by a microscope.

### 2.5. In Vitro Skin Insertion Tests

To evaluate the penetrating ability of MNs, the isolated porcine skin was taken out from −20 ℃ refrigerator and washed with 0.9 wt% normal saline solution before use, a process done to make sure the skin retained its elasticity and texture after defrosting. After the water on the skin surface was dried by filter paper, the porcine skin was placed in the PDMS block, and then the MNs were punctured into the skin for 5 min using thumb force. Next, the insertion region was recorded by a digital camera. The skin treated by MNs was also analyzed through frozen tissue sections technology. The frozen tissue was cut into 10 μm thick slides using freezing microtome (Lecia, Wetzlar, German) and then placed on adhesion microscope slides. The tissue slide was stained by hematoxylin and eosin (H&E) as well as being photographed under a microscope.

FITC was used as a model drug to further assess the drug permeation in skin. Following insertion into the porcine skin for 5 min, the skin was analyzed by confocal laser scanning microscope (CLSM, Carl Zeiss, Jena, German) using Z-stack mode. Scanning of the xy-plane was operated at an interval of 10 μm from the skin surface to inside via vertical direction. FITC was excited at 488 nm wavelength, with the emission wavelength being 525 nm. The depth of scanning was set from the skin surface to the plane where fluorescence disappeared.

### 2.6. In Vivo Dissolution of MNs after Insertion

For evaluation of in vivo dissolution of the MNs after insertion, the hair on the abdomen of SD rats was shaved before the experiment and methylene blue was loaded in the MNs for the ease of observation. During the experiment, the SD rats were anesthetized by 5% chloral hydrate, then the MNs were applied on the abdominal skin. MNs were removed from skin at predetermined intervals of 5, 10, and 20 min, respectively, which were observed by optical microscope to obtain the dissolved morphology of MNs.

### 2.7. In Vivo Skin Recovery after MNs Insertion

As a part of basic safety efforts, a preliminary skin recovery study was performed, in which methylene blue was loaded in the MNs in order to easily observe the skin irritation and recovery from MNs insertion. The hair on the abdomen of SD rats was depilated followed by the insertion of MNs into the abdominal skin, and MNs were then removed from the skin 5 min later. Photographs of the insertion area penetrated by MNs were taken by digital cinema after 0, 1, 2, 3, 6, and 12 h until blue micropore on the skin became invisible.

### 2.8. Drug Release from MNs

The drug release profile was evaluated by Franz diffusion cell (Kaikai Technology Trade Co., Ltd., Shanghai, China), in which the tips of the MNs were immersed into 8 mL 0.9 wt% normal saline solution in the receiver compartment, which was stirred at 200 rpm with the temperature set around 32 ± 0.5 °C. Sample (200 μL) was collected at 1, 3, 5, 10, 15, 20, 30, and 60 min from the bottom of the receiver compartment, respectively, and equal volume of fresh 0.9 wt% normal saline solution was supplemented immediately. The samples were diluted properly for analysis via HPLC.

### 2.9. In Vivo IVIS Image

The IVIS spectrum live imaging system (Perkin Elmer, Waltham, MA, USA) was used to assess the skin retention of drugs. Prior to the experiment, SD rats were anesthetized by 5% chloral hydrate, and the abdominal hair was depilated. Dissolving MNs loaded with both rhodamine B (20 μg) and propranolol hydrochloride (1.5 mg) were fabricated as described above, which were applied to the abdominal skin in SD rats for 10 min. For comparison, a rhodamine B solution (0.1%, 20 μL) was subcutaneously injected in the abdomen of an SD rat. The SD rats were imaged by IVIS at 30 min, 1, 2, 4, 6, 8, 10, 12, and 24 h after administration under Epi-Illumination mode with an excitation wavelength of 554 nm and emission wavelength of 580 nm.

### 2.10. Ex Vivo Skin Retention and Drug Permeation

Ex vivo drug permeation was evaluated using Franz diffusion cell with a diffusion area of 1.77 cm^2^. Full thickness backing skin of Bama porcine was collected for this study. The dissolving MNs array with a surface area of 0.64 cm^2^ containing 1.5 mg propranolol hydrochloride in the tip of the needles was inserted into the skin by thumb force, which was then fixed between the donor and acceptor compartment of the Franz diffusion cell. For comparison, a propranolol hydrochloride aqueous solution (1.5 mg/mL) was set as control, which applied 1 mL to either intact skin (solution-intact skin) or solid MNs to pretreated skin (solution-pretreated skin). The receiver fluid was 8 mL 0.9 wt% normal saline solution, which was stirred at 200 rpm. The temperature of the receiver fluid was controlled to get a skin surface temperature of 32 ± 0.5 °C at 1, 2, 4, 6, 9, and 12 h post-administration; samples were withdrawn from the receiver compartment and equal volume of fresh receiver fluid was added in the meantime. The samples were analyzed via HPLC.

The skin retention was also evaluated using the same procedure, except that the porcine skins were collected at 1, 2, 4, 6, 9, and 12 h post-administration. The skin was washed with 0.9 wt% normal saline solution three times to remove any propranolol hydrochloride that remained on the surface. Then the skin was cut into small pieces. Propranolol hydrochloride in the skin was extracted with methanol under ultrasonic for 20 min. The skin was then homogenized and centrifuged at 12,000 rpm for 30 min. After that, the supernatant was collected and centrifuged again at 8000 rpm for 30 min. Finally, the supernatant was diluted for analysis via HPLC. Skin drug concentration was recorded as µg/cm^2^.

### 2.11. HPLC Methods and Drug Loading

To quantify the propranolol hydrochloride content in MNs, the tip was first removed from the MNs patch and dissolved in 1 mL saline solution. The solution was vortexed for 5 min, followed by centrifuging for 10 min at 4000 rpm. The supernatant was collected and diluted appropriately for analysis via Agilent 1100 HPLC system (Agilent, Santa Clara, CA, USA).

Propranolol hydrochloride was separated by Agilent ZORBAX Eclipse XDB-C18 column (4.6 mm × 250 mm, 5 μm, CA, USA). The flow rate and the column temperature were set at 1 mL/min and 35 °C, respectively. The mobile phase was composed of equal volume of acetonitrile and water, which contained 1.6 g lauryl sodium sulfate and 0.1g NH_4_H_2_PO_4_ per 1000 mL. The detection wavelength was 290 nm with 10 μL injection volume. In the range of 1.563–50 μg/mL, a good linear relationship was found between the peak area (A) and the concentration (C) of propranolol hydrochloride, with a typical calibration curve of A = 11.575C−3.0378 (*R*^2^ =1). The extraction recovery rate, accuracy, and precision were all in line with biological sample analysis requirements.

### 2.12. Data Analysis

All data are expressed as mean ± standard deviation (SD), and all the experiments were repeated at least three times. Statistical analysis was conducted by SPSS (16.0) and one-way ANOVA was applied for groups comparison; a *p* value < 0.05 was considered statistically significant.

## 3. Results and Discussion

### 3.1. Morphology of the MNs and Drug Distribution

**Figure 2:**
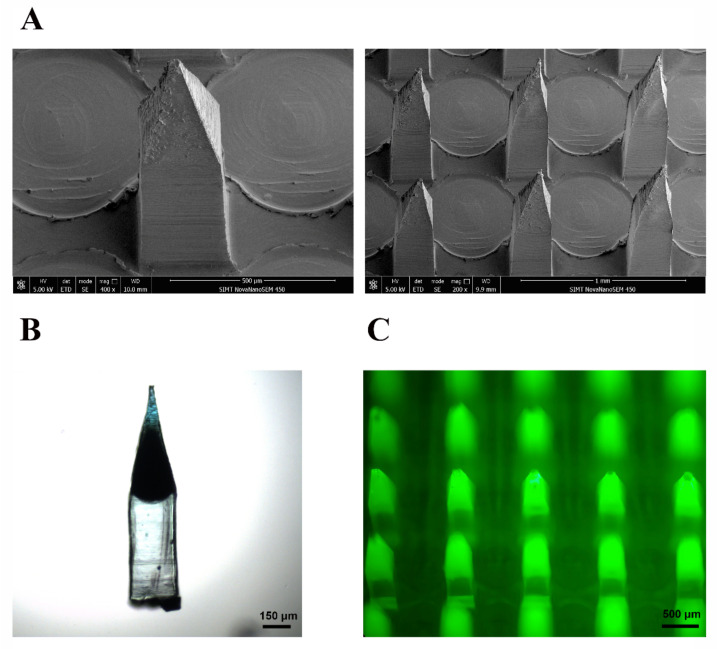
Characterization of the microneedles. (**A**) Scanning electron microscopy images show the morphology of the microneedles. (**B**) Drug distribution in the tip of the needle was illustrated by methylene blue. (**C**) Fluorescence images of the microneedles with FITC as a tracer reagent.

In this study, HA and PVP-K90 were used as matrix materials to produce MNs. HA is a natural linear polysaccharide with excellent water solubility, biocompatibility, biodegradability, and mechanical properties, which has been used extensively in the fabrication of MN [58,59,60]. However, dissolving MNs prepared only by HA have high fragility, whereas PVP-K90 is a polymer with good toughness and hardness [30,61,62]. Thus, PVP-K90 was used to adjust the mechanical strength and fragility of the MNs. Figure 2A shows the morphology of dissolving MNs. The needles present obelisks, being 1200 μm in height with a side length of 300 μm in the base. The shape proved to be better in penetration and higher in cutaneous delivery efficiency than pyramidal or circular ones [63]. The needles were arranged in a 12 × 12 array in a patch of 8 mm × 8 mm with a center-to-center interval of 600 μm. Furthermore, the two-step procedure of casting was adopted for loading drugs in the tips of the needles [64,65]. As shown in Figure 2B,C, methylene blue and FITC were primarily dispersed in the tips of the needles. This fabrication method could avoid drug waste during preparation and application. Furthermore, there was no need to insert full-length needles, which could reduce the stimulation of the MNs to the nerve and reduce pain.

### 3.2. The Mechanical Strength of the MNs

**Figure 3:**
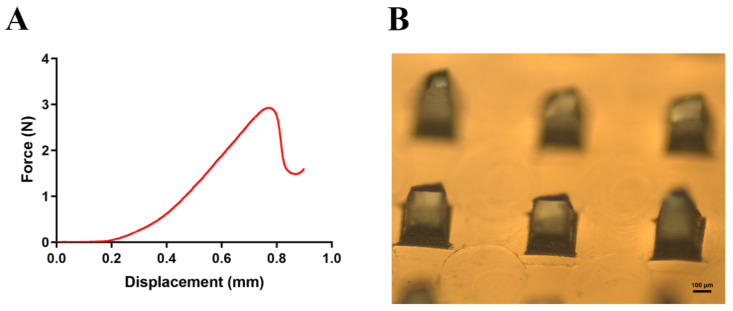
(**A**) The force-displacement curve of the microneedles. (**B**) The morphology of the microneedles treated by texture analyzer when the force dropped sharply.

The needles should be strong enough to puncture the skin [66,67]. In this experiment, the mechanical strength was tested by texture analyzer in vitro. The pressure force gradually increased with the increased displacement in the beginning and significantly increased when the displacement exceeded 0.2 mm. The force dropped sharply at the displacement exceeding 0.80 mm (Figure 3A). We observed the morphology of the MNs when the force dropped sharply (Figure 3B), indicating broken tips. We assumed that the MNs broke at the point of sudden drop in pressure, which was defined as the failure force of MNs [67]. According to the previous study, MNs could be successfully inserted into skin without breaking when the failure force is greater than 0.24 N/needle [68,69]. The resultant failure force of dissolving MNs in this experiment could be quantified as 3 N/needle, proving that MNs provided enough strength to pierce skin.

### 3.3. In Vitro Skin Insertion Tests

**Figure 4:**
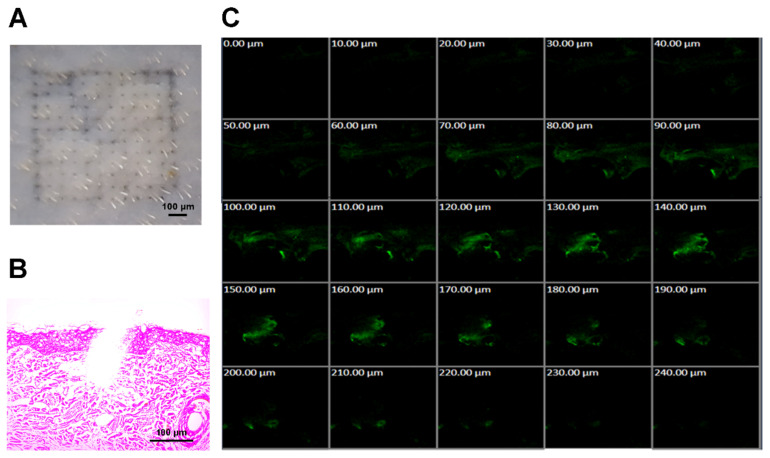
Skin insertion capability of the microneedles. (**A**) Digital camera image of porcine skin treated by microneedles. (**B**) H&E staining of the skin treated by the microneedles. (**C**) CLSM photos of porcine skin treated by FITC-loaded microneedles.

To further evaluate the penetrating ability of MNs, dissolving MNs were applied into full thickness porcine skin for 5 min and then removed. As shown in Figure 4A, the micropores in the skin indicated that needles were inserted into the skin and dissolved successfully. The skin was further analyzed by H&E staining. The results confirmed that the needles could pierce the SC and reach the dermis (Figure 4B). However, despite the length of 1200 μm, the needles only penetrated 200–300 μm of the skin, resulting from the existing wrinkling of the skin [70]. Since the drugs were loaded in the tips of the needles, the penetration depth was deep enough to release all of the cargo. In order to confirm this estimation, FITC was loaded in the tips of the MNs to mimic the drugs. Following the same procedure of administration, the skin was observed under the CLSM (Figure 4C). The FITC fluorescence was observed till the depth of 220 μm beneath the skin surface, while the signals mainly resided from the depth of 50 μm to 220 μm and presented a narrowing trend. The results were in accordance with those from the tissue section. In addition, the FITC fluorescence was faint in the upper parts of the skin from the surface to the depth of 40 μm due to the tip loading design. Therefore, it was confirmed that the needles could penetrate the SC and deliver all of the cargo into the skin.

### 3.4. In Vivo Dissolution of the MNs after Insertion and the Recovery of the Skin

**Figure 5:**
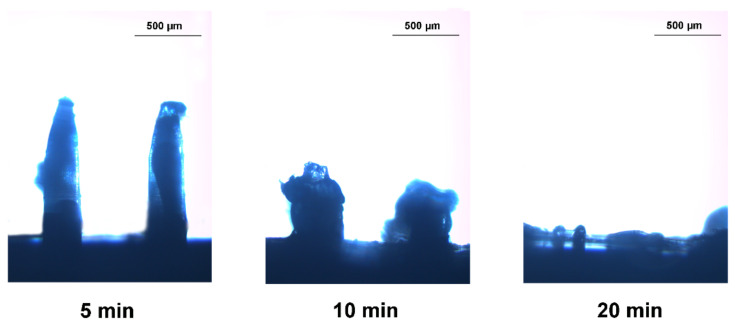
Morphological changes of the needles after insertion into the abdominal skin of SD rat at 5, 10, and 20 min.

The dissolution of the MNs in vivo was evaluated in abdominal skin of SD rats. For the convenience of observation, methylene blue was used to stain the MNs. The morphological changes of MNs after insertion into the skin for 5 min, 10 min, and 20 min were recorded by using optical microscopy, respectively. At 5 min post administration, the blue of the needles was obviously lighter than that of the base, indicating the dissolution of the needle matrix; at 10 min post administration, the needles dissolved more than 50% and dissolved almost totally within 20 min (Figure 5). The results illustrate that the needles composed of HA and PVP-K90 could dissolve quickly after piercing.

### 3.5. In Vivo Skin Recovery after MNs Insertion

**Figure 6:**
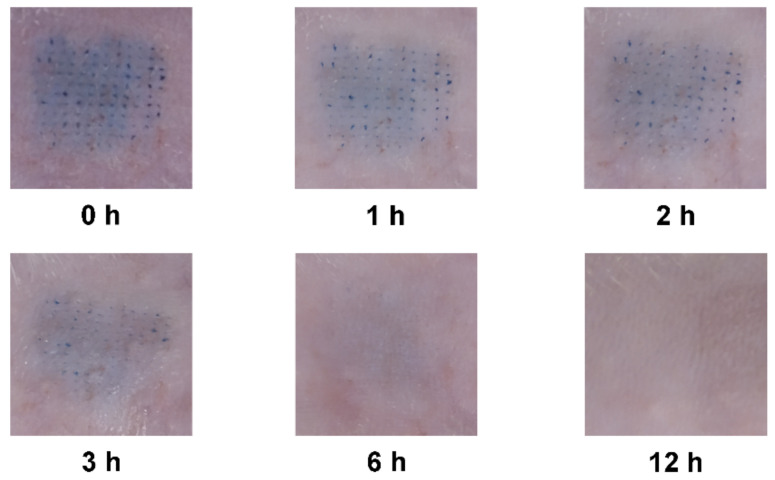
The recovery of abdominal skin in SD rat after treatment by the microneedles.

The recovery of the abdominal skin of the SD rat from the treatment of methylene blue loaded MNs was evaluated via observation with a digital camera (Figure 6). The MNs treatment did not induce obvious adverse effects except skin irritation and a trace of interstitial fluid exudate, which were recovered rapidly. The micropores created by MNs almost faded away at 3 h postinsertion. Only faint blue was left in the skin, and almost disappeared within 12 h.

### 3.6. Loading of Propranolol Hydrochloride in the MNs

**Figure 7:**
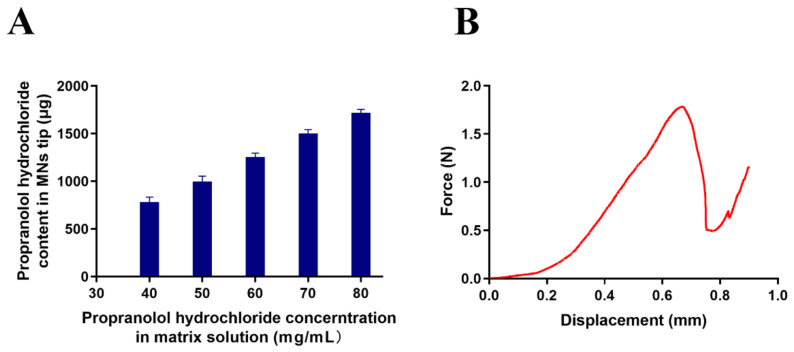
(**A**) Content of propranolol hydrochloride in the MNs was linearly increased with the increase of its content in the tip solution (*n* = 3). (**B**) The force-displacement curve of propranolol hydrochloride-loaded dissolving MNs patch.

The previous results indicated that HA and PVP-K90 matrix provides excellent mechanical strength for skin penetration without compromising the dissolution. The two-step procedure of casting deposited the cargo in the tips and enabled delivery of all dose into the skin. Then we tried to load propranolol hydrochloride in the MNs using the same procedure, except that propranolol hydrochloride was dissolved in the tip solution. To investigate the loading capacity of the MNs, the concentration of propranolol hydrochloride in the tip solution was increased from 40 mg/mL to 80 mg/mL at equal intervals of 10 mg/mL. The contents of propranolol hydrochloride in the corresponding microneedles were linearly increased from 781.90 ± 51.85 μg to 1718.65 ± 34.92 μg (Figure 7A). We didn’t further increase the loading of propranolol hydrochloride because the content of 1500 μg was adequate. Furthermore, the mechanical strength of the needles loading propranolol hydrochloride still maintained enough strength to puncture into skin, which was 1.7 N/needle at propranolol hydrochloride content of 1718.65 ± 34.92 μg, as shown in Figure 7B.

### 3.7. Drug Release from MNs

**Figure 8:**
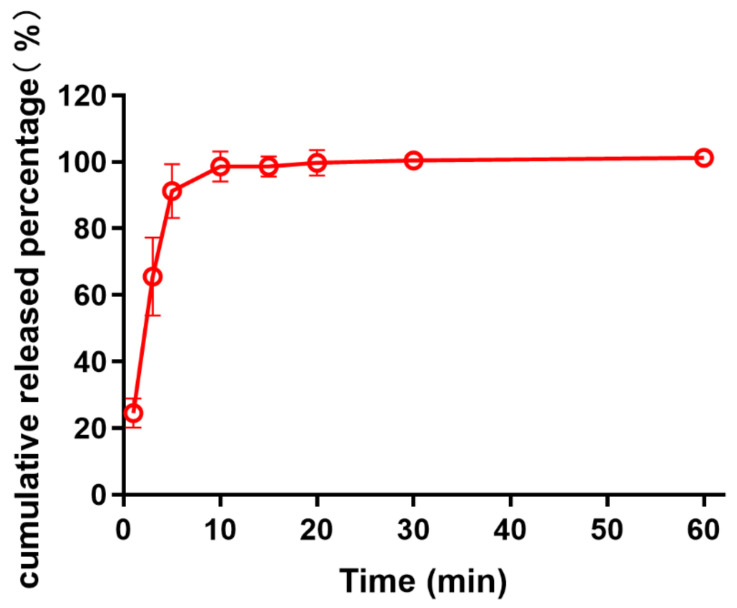
In vitro cumulative release percentage of propranolol hydrochloride from dissolving MNs (*n* = 3).

In this experiment, drug release was conducted at pH 7.4 under sink conditions. Owing to the excellent water solubility of HA and PVP-K90, there was an initial burst of drug release within 10 min, in which the cumulative released percentage could be up to 90%, as shown in Figure 8. Afterward, the release rate gradually slowed down; approximately 100% amount of propranolol hydrochloride was released into the receiver medium at 20 min, and the cumulative amount almost remained unchanged from 20 min to 1 h. In addition, the cumulative propranolol hydrochloride release was almost equivalent to the drug content in MNs, which presumably means that the matrix materials had no interference in drug release from MNs.

### 3.8. In Vivo IVIS Image

**Figure 9:**
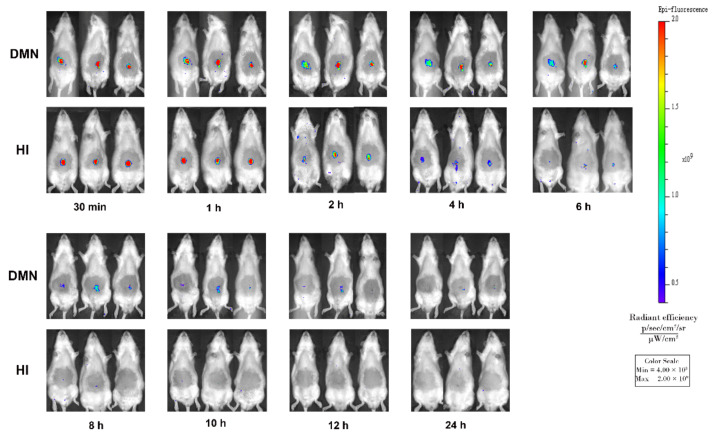
In vivo fluorescence intensity images of SD rats after application of rhodamine B via dissolving MNs or hypodermic injected (*n* = 3).

Furthermore, drug diffusion into skin after MNs application was determined by IVIS with rhodamine B being a model drug because of its similar property to propranolol hydrochloride: a small molecule with good water solubility. Meanwhile, rhodamine B solution was hypodermic injected (HI) as a control. The IVIS images at different times are shown in Figure 9. It was found that fluorescence signals in abdominal skin of SD rats were intense after MNs treatment and were sustained for 12 h. In contrast, fluorescence signals disappeared quickly in the HI group. The results demonstrated that MNs formed by HA and PVP-K90 could quickly dissolve via absorbing the interstitial fluid, and drugs were then gradually diffused into the skin from the dissolved matrix. Thus, the skin area with a high local drug concentration became a drug depot to further release the drug continuously for nearly 12 h. The prolonged drug retention may be associated with the material used in the dissolving MNs’ fabrication. It was previously reported that hydration of the epidermis induced by HA could be beneficial for drug skin retention and slow permeation [59,71].

### 3.9. Permeation and Skin Retention

**Figure 10:**
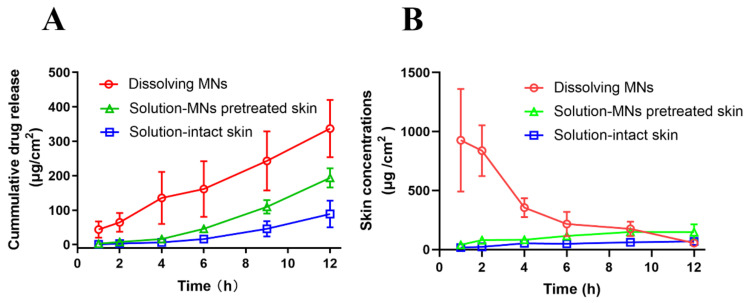
(**A**) Permeation profiles of propranolol hydrochloride into a receiver fluid after application of propranolol hydrochloride in different approaches to full thickness excised porcine skin (*n* = 5). (**B**) Skin concentration of propranolol hydrochloride after application of propranolol hydrochloride in different approaches to full thick excised porcine skin (*n* = 3). The amount of drug was 1.5 mg.

Many previous studies have confirmed that dissolving MNs could be utilized as an efficient tool for improving the penetration rate and delivery efficiency of an encapsulated drug [35,72,73]. In this experiment, full thickness isolated porcine skin was used as a drug permeable membrane. The permeation profiles of propranolol hydrochloride from different approaches are shown in Figure 10. The solution that was applied to the intact skin showed inferior permeability to the other two groups due to the hydrophilicity of propranolol hydrochloride. The permeated amounts of propranolol hydrochloride in this group were the lowest among all groups at all time points. Pretreatment with solid MNs created micropores in the skin, facilitating diffusion of propranolol hydrochloride from outside the skin into the skin. Therefore, the aqueous solution applied to the pretreated skin displayed accelerated penetration, which is shown by the continuously increasing slope of the curve (Figure 10A). However, although pretreatment with solid MNs could create micropores in the skin for diffusion of propranolol hydrochloride, the lipids that reside among the coenocytes may still delay the diffusion of hydrophilic molecules such as propranolol hydrochloride [74,75]. By contrast, the dissolving MNs directly delivered propranolol hydrochloride into the skin, while the needle matrix dissolved rapidly. The absence of the diffusion step from outside the skin into the skin endowed even faster permeation of propranolol hydrochloride from the dissolving MNs than the solution that was applied to the pretreated skin. Thus, compared with the solution counterpart, irrespective of being applied to intact or pretreated skin, the dissolving MNs showed significantly higher permeating amounts of propranolol hydrochloride at all time points.

Furthermore, the skin retention of propranolol hydrochloride was also evaluated. It is necessary to establish a method for determining the drug content in the skin for comparison, which will be mandatory for the optimization of transdermal medicinal formulations. Skin concentrations of propranolol hydrochloride at different times were tested and are shown in Figure 10B. The result demonstrates that skin concentrations in the dissolving MNs group were higher than those in the MN-pretreated group or control group, and propranolol hydrochloride could be detected in the skin within 12 h. After MNs insertion, HA and PVP-K90 dissolved by absorbing interstitial fluid to form a high local drug concentration at the beginning and then released drug continuously. Therefore, dissolving MNs may contribute to improved administration regimens by increasing the concentration of the drug in the local skin and decreasing dosing frequency.

## 4. Conclusions

Oral propranolol for the treatment of IHs may have potential adverse effects due to systemic absorption. Dermal administration of propranolol could increase the local concentration of the drug, reduce the frequency of administration, and improve patient compliance. Propranolol hydrochloride loaded microneedles were fabricated in this study. The matrix composed of HA and PVP-K90 provided excellent mechanical strength for piercing the skin and dissolved quickly when absorbing interstitial fluid after insertion. Propranolol hydrochloride was primarily concentrated in the tip via a two-step procedure. Most important, the cumulative permeation and skin retention of propranolol hydrochloride in dissolving MNs treated skin were much higher than those of propranolol hydrochloride solution applied in blank skin and solid MNs treated skin. Furthermore, the benefit of increasing drug skin retention induced by HA-mediated skin hydration is that the skin can act as a drug repository, allowing continuous release of the drug. Taken together, dissolving MNs made from HA and PVP-K90 display great advantages for transdermal delivery. Nevertheless, further animal experiments are necessary to evaluate the pharmacokinetic and pharmacodynamic profiles of the MNs for successful application.

## Data Availability

Not applicable.
